# Predictive factors of bleeding events in adults undergoing extracorporeal membrane oxygenation

**DOI:** 10.1186/s13613-016-0196-7

**Published:** 2016-10-06

**Authors:** Cécile Aubron, Joris DePuydt, François Belon, Michael Bailey, Matthieu Schmidt, Jayne Sheldrake, Deirdre Murphy, Carlos Scheinkestel, D Jamie Cooper, Gilles Capellier, Vincent Pellegrino, David Pilcher, Zoe McQuilten

**Affiliations:** 1 The Australian and New Zealand Intensive Care Research Centre, Department of Epidemiology and Preventive Medicine, Monash University, Melbourne, Australia; 2 The Transfusion Research Unit, Department of Epidemiology and Preventive Medicine, Monash University, Melbourne, Australia; 3 The Intensive Care Unit, The Alfred Hospital, Melbourne, Australia; 4 University Hospital of Antwerp, 2000 Antwerp, Belgium; 5 The Intensive Care Unit, Jean Minjoz Hospital, 25030 Besançon, France; 6 School of Public Health and Preventive Medicine, Monash University, Melbourne, Australia; 7 Medical Intensive Care Unit, Centre Hospitalier Régional et Universitaire de Brest, site La Cavale Blanche, Bvd Tanguy Prigent, 29609 Brest Cedex, France; 8 LUBEM, EA 3882 - Université de Bretagne Occidentale, 29 200 Brest, France; 9 Medical Intensive Care Unit, iCAN, Institute of Cardiometabolism and Nutrition, Hôpital de la Pitié–Salpetrière, Assistance Publique–Hôpitaux de Paris, Paris, France

**Keywords:** Extra corporeal membrane oxygenation, Critically ill patients, Haemorrhage, Bleeding, Anticoagulation, Coagulopathy, Thromboembolic events

## Abstract

**Background:**

Bleeding is the most frequent complication associated with extracorporeal membrane oxygenation (ECMO) support in critically ill patients. Nonetheless, risk factors for bleeding have been poorly described especially those associated with coagulation anomalies and anticoagulant therapy during ECMO support. The aim of this study is to describe bleeding complications in critically ill patients undergoing ECMO and to identify risk factors for bleeding events.

**Methods:**

We retrospectively analysed ICU charts of adults who received either veno-venous (VV) or veno-arterial (VA) ECMO support in two participating ICUs between 2010 and 2013. Characteristics of patients with and without bleeding complications, as per the Extracorporeal Life Support Organisation (ELSO) definition, were compared, and the impact of bleeding complications on patient outcomes was assessed using survival analysis. Variables that were independently associated with bleeding, including daily clinical and biological variables during ECMO courses, were modelled.

**Results:**

Of the 149 ECMO episodes (111 VA ECMO and 38 VV ECMO) performed in 147 adults, 89 episodes (60 %) were complicated by at least one bleeding event. The most common bleeding sources were: ECMO cannula (37 %), haemothorax or cardiac tamponade (17 %) and ear–nose and throat (16 %). Intra-cranial haemorrhage occurred in five (2.2 %) patients. Bleeding complications were independently associated with worse survival [adjusted hazard ratio (HR) 2.17, 95 % confidence interval (CI) 1.07–4.41, *P* = 0.03]. Higher activated partial thromboplastin time (aPTT) [adjusted odds ratio (OR) 3.00, 95 % CI 1.64–5.47, *P* < 0.01], APACHE III score [adjusted OR 1.01, 95 % CI 1.01–1.02, *P* = 0.01] and ECMO following surgery [adjusted OR 3.04, 95 % CI 1.62–5.69, *P* < 0.01] were independently associated with greater risk of bleeding occurrence. A similar association between bleeding and higher aPTT was found when non-post-surgical VA ECMO was considered separately.

**Conclusions:**

Bleeding events based on the ELSO bleeding definition occurred in more than 60 % of ECMO episodes and were associated with hospital mortality. We identified higher aPTT prior bleeding as an independent risk factor for bleeding event, suggesting that better control of the aPTT (through a better control of either coagulopathy or anticoagulation) may improve patients’ outcome.

## Background

Bleeding is one of the most frequent complications occurring in patients undergoing extracorporeal membrane oxygenation (ECMO), occurring in approximately 30 % of patients receiving ECMO therapy [1–4]. Bleeding events independently impact on patient prognosis, including mortality [1, 2]. This association may be explained by the site of bleeding, with intra-cranial haemorrhage (ICH) identified in more than 40 % of non-survivors in patients treated with ECMO during the H1N1 flu outbreak [5].

Many factors that may place patients undergoing ECMO at higher risk of bleeding have been identified [6], and these can be classified into patient, treatment and circuit related. The systemic inflammatory response syndrome (SIRS) and contact between the patient’s blood with the ECMO circuit lead to activation of the coagulation cascade, with effects on fibrinolysis, thrombin formation and platelet function [6–8]. The results of these changes to haemostatic balance may be difficult to predict, with the coexistence of both thrombotic and haemorrhagic risks. Although use of bioline surface-heparinised extracorporeal perfusion circuits reduces the risk of thrombosis, with decreases in inflammation, coagulation activation and platelet consumption, anticoagulation remains standard practice in patients undergoing ECMO to offset circuit-associated thrombotic risks [9, 10]. Indeed, thrombotic events might complicate ECMO therapy with significant morbidity and mortality. Thrombotic events have been identified in approximately 15 % of ECMO courses [11], but they are likely to be underdiagnosed and underreported [12].

A better knowledge of characteristics of bleeding during ECMO is essential to improve management and outcomes of patients undergoing ECMO. Studies on risk factors for haemorrhagic complications have predominantly been performed in paediatric patients or suffer limitations, including lack of standard bleeding definition and the timing of the study with many advances in ECMO circuit and care over the past decades [13, 14].

Therefore, we aimed to study bleeding complications in critically ill patients undergoing ECMO to define incidence, consequences on patient prognosis and factors associated with increased bleeding risk.

## Patients and methods

### Study design

We conducted a retrospective study in two centres, the Alfred Hospital (Melbourne, Australia) and the Hospital of Besançon (France). The Alfred Hospital is a teaching hospital affiliated to Monash University, which provides heart and lung transplantation services for the states of Victoria, South Australia and Tasmania. It has an intensive care unit (ICU) with a 45-bed capacity and is the referral centre for adult trauma and ECMO in Victoria. The Hospital of Besançon is a teaching hospital affiliated to the University of Franche-Comté; its 21-bed medical ICU has long-standing experience in ECMO support.

All adults admitted to ICU between January 2010 and June 2013 at the Alfred Hospital and between January 2013 and December 2013 at the Hospital of Besançon and who underwent ECMO were included.

The study was approved by the Alfred Health Human Research Ethics Committee (202/11) and by the Ethics Committee of Besançon Hospital.

### ECMO haemostatic practice

Both hospitals had anticoagulation protocols and local guidelines for haemostatic management for patients undergoing ECMO, and these protocols were similar for VV and VA ECMO. In both centres, systemic anticoagulation with a heparin infusion targeting an activated partial thromboplastin time (aPTT) between 50 and 70 s was standard practice, unless patients were bleeding or at increased risk of bleeding (usually related to peri-operative and traumatic injuries). Red blood cell (RBC) transfusion was given to maintain a haemoglobin (Hb) concentration above 8 g/dL. Prophylactic blood products were not routinely given for coagulation abnormalities, with the exception of severe thrombocytopenia (<50,000 platelets/mm^3^). In the case of bleeding, platelets were administered to maintain a platelet count ≥80,000/mm^3^, fresh frozen plasma (FFP) to maintain the international normalised ratio (INR) ≤1.5 or prothrombin (PT) >70 %, and cryoprecipitate or fibrinogen concentrate to maintain fibrinogen plasma concentration ≥1.5 g/L. Coagulation factor concentrates, antifibrinolytic agents (tranexamic acid), antithrombin III and protamine were administered when deemed appropriate by the intensivist in charge. Heparin was the anticoagulant of choice; however, alternatives (warfarin, lepirudin) were used if necessary. ECMO circuits were phosphorylcholine and heparin bonded in both centres. Circuit membrane was changed at the Alfred Hospital if there was evidence of systemic fibrinolysis presumed to be due to circuit or clot in the oxygenator and at both sites in cases of poor oxygenator function and increases in trans-oxygenator pressure. Proton pump inhibitors were routinely administered to ECMO patients. At the Alfred Hospital, both cannulae of VA and VV ECMO were percutaneously inserted, while at Hospital of Besançon cannula of VA ECMO was surgically inserted.

### Clinical and biological data

Medical history and clinical charts were retrospectively reviewed, and the following data were collected: demographics, comorbidities, ICU and hospital admission and discharge dates, diagnosis and Acute Physiology and Chronic Health Evaluation (APACHE III) score at admission. Treatment with aspirin, clopidogrel or vitamin K antagonist, surgery or cardiopulmonary resuscitation prior to ECMO initiation was recorded. The Sequential Organ Failure Assessment (SOFA) scores prior to ECMO initiation and on the third day of ECMO were calculated. Requirement for renal replacement therapy (RRT), mechanical ventilation (MV) or intra-aortic balloon pump while on ECMO were also collected. The daily highest and lowest values of the following biological data were recorded for each day on ECMO: Hb on formal full blood examination, platelet count, aPTT, INR, PT and fibrinogen level. The lowest temperature, the lowest value of arterial pH and ionised calcium, the highest D-dimer, urea, bilirubin and free Hb were also collected for each day on ECMO.

### ECMO data

ECMO data included the commencement and cessation date, whether the ECMO was initiated in another hospital, the main indication for ECMO and the type of ECMO [veno-venous (VV) or veno-arterial (VA) including central and peripheral VA ECMO]. The following data were also recorded: outcomes of ECMO, status at ICU and hospital discharge and location of hospital discharge.

### Bleeding events and thromboembolic events

Daily information on blood product and haemostatic agent use, including type and dose of anticoagulant, was collected for the duration of ECMO.

Bleeding events were defined according to the Extracorporeal Life Support Organisation (ELSO) definition [15]: we defined a bleeding event if there was clinically overt bleeding recorded in the medical and/or nursing charts associated with either administration of 2 or more RBC units in 24 h or a drop in haemoglobin greater than 2 g/L over 24 h, or if there was a haemothorax, central nervous system or retroperitoneal bleeding, or if bleeding required an intervention. If there were consecutive days with the same primary source of bleeding as preceding days, these were considered the same bleeding event. When a patient had more than one bleeding source on the same day, this was also recorded.

Thromboembolic complications, including deep venous thrombosis, ischaemic stroke, intra-cardiac thrombus, pulmonary embolism and membrane circuit clotting requiring membrane change, were collected from the medical record.

### Statistical analysis

All analyses were performed using Stata 12.0 (College Station, Texas). Descriptive statistics are reported as mean (standard deviation) or median (inter-quartile range, IQR) according to data distribution. Hypothesis testing for patient level data was performed using Chi-square for categorical variables, Student’s *t* test for normally distributed data and Wilcoxon’s rank sum for non-normally distributed data. For comparisons between ECMO days with and without bleeding, to account for the repeated measures per patient (with bleeding recorded on each day of ECMO treatment), a repeated measures mixed model was performed for continuous variables and random effects logistic model for binomial variables. Mean and 95 % confidence interval (CI) are reported adjusted for repeated measures.

Survival curves were plotted using the Kaplan–Meier method, and groups were compared using the log-rank test. Multivariable analysis for predictors of survival was performed using a Cox proportional hazard regression model, including those variables that were associated with the outcome with a *P* < 0.2. As the relationship between bleeding and survival was time dependent (with bleeding occurring at different times during the course of ECMO treatment), bleeding variables were treated as time-varying covariates in the Cox proportional hazard models. The final models were assessed for proportionality using the proportional hazards assumption test.

To investigate predictors of bleeding, multiple logistic regression modelling was performed. Initially, a backward stepwise logistic model was performed, which only included variables recorded prior to the day of bleeding (e.g. highest aPTT on the day prior to the bleeding event, anticoagulation on the day prior to the bleeding event, etc.). aPTT was categorised into quartiles for the multivariable analysis. To account for the repeated measures per patient, variables that were found to be independently associated with the bleeding outcomes in the stepwise logistic model were then included in a multi-level logistic regression model, with each patient now modelled as a random effect. Subgroup analyses were performed in patients undergoing non-post-surgical VA ECMO.

## Results

Over the study period, 149 episodes of ECMO support, 38 VV and 111 VA ECMO, were conducted in both hospitals (Alfred, *n* = 125, and Besançon, *n* = 24). The median duration of support was 8 days (first and third quartiles: 5–14) for the VV ECMO and 6 days (first and third quartiles: 4–10) for the VA ECMO. Characteristics of study cohort are given in Table 1.

**Table 1:** Comparison of ECMO episodes with and without bleeding events

Variable	All episodes (*n* = 149)	No bleeding (*n* = 60)	Bleeding (*n* = 89)	*P*
Age (year ± SD)	46.5 ± 14.9	44.5 ± 15.1	47.8 ± 14.7	0.18
Male sex	92 (62 %)	38 (63 %)	54 (61 %)	0.74
Weight (kg ± SD)	80.7 ± 22	80.7 ± 22	80.7 ± 22	1.00
APACHE III score ± SD	77.3 ± 33.6	70.1 ± 30	82.1 ± 35.2	0.03
*Comorbidities*				
Immunosuppressed	34 (23 %)	8 (14 %)	26 (29 %)	0.027
Hepatic failure	3 (2 %)	2 (3 %)	1 (1 %)	0.34
Cirrhosis liver disease	2 (1.3 %)	0 (0 %)	2 (2 %)	0.25
Insulin dependent diabetes	7 (5 %)	3 (5 %)	4 (5 %)	0.87
Chronic respiratory failure	23 (16 %)	4 (7 %)	19 (21 %)	0.018
Chronic cardiovascular disease	38 (26 %)	14 (24 %)	24 (27 %)	0.66
Chronic renal failure	2 (1.3 %)	0 (0 %)	2 (2 %)	0.25
*Indications for ECMO*				
Acute cardiomyopathy	20 (13 %)	12 (20 %)	8 (9 %)	0.05
AMI	28 (19 %)	9 (15 %)	19 (21 %)	0.33
Chronic cardiomyopathy	14 (9 %)	8 (13 %)	6 (7 %)	0.18
Heart transplant	16 (11 %)	4 (7 %)	12 (13 %)	0.19
Lung transplant	15 (10 %)	3 (5 %)	12 (13 %)	0.09
Pneumonia	27 (18 %)	16 (27 %)	11 (12 %)	0.03
Post CAGS or valve surgery	9 (6 %)	1 (2 %)	8 (9 %)	0.07
Other	20 (13 %)	7 (12 %)	13 (15 %)	0.61
Post-surgical ECMO	39 (26 %)	4 (7 %)	35 (39 %)	<0.001
Transplantation prior	33 (22 %)	8 (13 %)	25 (28 %)	0.033
Cardiac arrest before	39 (26 %)	14 (23 %)	25 (28 %)	0.52
*Medication prior ECMO*				
Aspirin	35 (24 %)	13 (22 %)	22 (26 %)	0.59
Clopidogrel	17 (12 %)	5 (8 %)	12 (14 %)	0.32
Warfarin	16 (11 %)	4 (7 %)	12 (14 %)	0.16
ECMO started in another hospital	40 (27 %)	20 (33 %)	20 (22 %)	0.14
*ECMO type*				
VA ECMO	111 (74 %)	40 (67 %)	71 (80 %)	0.07
VV ECMO	38 (26 %)	20 (33 %)	18 (20 %)	0.07
Days on support, median [IQR]	7 [5–11]	6 [5–10]	8 [5–12]	0.09
Centre Alfred Hospital	128 (86 %)	52 (87 %)	76 (85 %)	0.83
RRT at any time	60 (43 %)	18 (31 %)	42 (51 %)	0.02
MV at any time	145 (97 %)	57 (95 %)	88 (99 %)	0.15
*SOFA score prior ECMO median [IQR]*				
Total	10 [7–13]	9 [7–12]	11 [9–14]	0.01
SOFA respiratory	3 [2–4]	3 [2– 4]	3 [1–4]	0.95
SOFA coagulation	0 [0–1]	0 [0–1]	1 [0–2]	0.17
SOFA liver	0 [0–2]	0 [0–1]	1 [0–2]	0.02
SOFA cardiovascular	4 [4–4]	4 [3–4]	4 [4–4]	0.02
SOFA neurology	1 [0–2]	0 [0–1]	1 [0–3]	0.02
SOFA renal	1 [0–3]	1 [0– 2]	1 [0–3]	0.21
*Blood product use during ECMO*				
Median RBC unit (IQR)	6 [2–14]	2 [0.5–4]	12 [6–19]	<0.01
Median PLT doses (IQR)	1 [0–3]	0 [0–1]	2 [0–5]	<0.01
Median FFP unit (IQR)	2 [0–6]	0 [0–1]	5 [1–10]	<0.01
Median cryoprecipitate (IQR)	0 [0–0]	0 [0–0]	0 [0–1]	<0.01
*Other adverse events*				
Ischaemic stroke	4 (2.7 %)	1 (2 %)	3 (3 %)	0.53
Membrane change	16 (11 %)	6 (10 %)	10 (11 %)	0.81
Limb ischaemia any stage	11 (7 %)	2 (3 %)	9 (10 %)	0.12
*Outcomes*				
Weaned off ECMO	95 (64 %)	42 (70 %)	53 (60 %)	0.19
Never weaned	36 (24 %)	10 (17 %)	26 (29 %)	0.08
Bridge to other assistance	18 (12 %)	8 (13 %)	10 (11 %)	0.70
Median (IQR) ICU LOS (days)	17 [9–28]	13.5 [8–23]	16 [9–28]	0.18
Median (IQR) hospital LOS (days)	36.5 [15–56]	27.5 [13.5–36.5]	36.5 [15–56]	0.04
ICU status, alive	101 (68 %)	45 (75 %)	56 (63 %)	0.12
Hospital status, alive	99 (66 %)	45 (75 %)	54 (61 %)	0.07
Discharge home	65 (66 %)	33 (55 %)	32 (36 %)	0.02

Data presented as *n* (%) categorical variables and median (interquartile range) for nonparametric variables

“Post-surgical ECMO” includes any surgery (CAGS and other)

*ECMO* extracorporeal membrane oxygenation, *APACHE III* score Acute Physiology and Chronic Health Evaluation III score, *AMI* acute myocardial infarction, *CAGS* coronary artery graft surgery, *VA ECMO* veno-arterial ECMO, *VV ECMO* veno-venous ECMO, *RRT* renal replacement therapy, *MV* mechanical ventilation, *SOFA* Sequential Organ Failure Assessment, *RBC* red blood cell unit, *FFP* fresh frozen plasma, *PLT* platelets bag, *ICU* intensive care unit, *LOS* length of stay

### Bleeding events

**Fig. 1:**
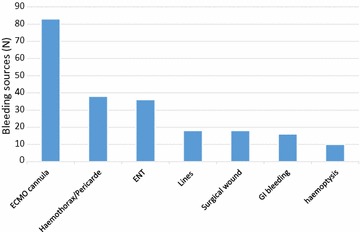
Bleeding sources. Results are expressed as absolute values (*n*). There were 224 sources identified in 128 haemorrhage events. *Lines* include central venous catheter; *ENT* ear–nose and throat, *GI* gastrointestinal

Overall there were 128 bleeding events (with a bleeding event defined as consecutive days meeting the bleeding definition with the same source of bleeding) over 203 ECMO days with 224 bleeding sources identified. Of the 149 ECMO episodes, 58 (39 %) had one, 14 (9 %) had two, 9 (6 %) had three, 5 (3 %) had four and 3 (2 %) had five bleeding events during the ECMO course. The median time to the first bleeding event from starting ECMO was 4 days (first and third quartiles: 0–8 days). Of the 224 sources of bleeding identified, the three most frequent were ECMO cannula, post-cardiothoracic surgical haemothorax or tamponade, and ear–nose and throat (ENT) haemorrhage (Fig. 1). Intra-cranial haemorrhage (ICH) occurred in 5 patients (2.2 %) and was fatal in all cases. There were 67 specific haemostatic interventions for bleeding in 43 ECMO episodes that included surgical intervention (*n* = 56), angiography (*n* = 5), gastroscopy (*n* = 4) and bronchoscopy (*n* = 2). Tranexamic acid was administered in 15 bleeding events, activated recombinant factor VII in 3 events and prothrombin complex concentrate and fibrinogen concentrate in 5 and 2 bleeding events, respectively.

Eighty-nine ECMO episodes (60 %) were complicated by at least one bleeding event. There were no differences in patient demographics and comorbidities between those who experienced bleeding complications and those who did not (Table 1). When haemorrhagic complications occurred during ECMO, patients were more likely to have surgery prior to ECMO (39 vs. 7 %, *P* < 0.001), had greater illness severity at ECMO initiation with a median SOFA score of 11 (first and third quartiles: 9–14) vs. 9 (first and third quartiles: 7–12) (*P* = 0.01) and more often required RRT (64 vs. 35 %; *P* < 0.01). Antiplatelet therapy or warfarin prior to ECMO was not associated with bleeding events in univariate analysis, nor was the duration of ECMO.

When considering the days on ECMO individually, 1144 days on ECMO were free of bleeding while 203 met the ELSO definition of bleeding. Lower Hb [mean 87 g/L (95 % CI 84–89) vs. 93 g/L (95 % CI 90–95); *P* < 0.01], lower arterial pH [mean 7.30 (95 % CI 7.28–7.32) vs. 7.35 (95 % CI 7.33–7.36); *P* < 0.01], lower ionised calcium [mean 1.02 mmol/L (95 % CI 1.01–1.05) vs. 1.09 mmol/L (95 % CI 1.07–1.10); *P* < 0.01] and higher aPTT [mean 90 s (95 % CI 81–98) vs. 71 s (95 % CI 67–76); *P* < 0.01] recorded on the day prior to bleeding were associated with bleeding occurrence when considering all patients with and without heparin (Table 2). A lower proportion of patients with bleeding had received heparin on the previous day compared to those without bleeding; however, those who did receive it and who were bleeding had higher aPTT compared to the non-bleeding patients [mean 86 s (95 % CI 76–95) vs. 69 s (95 % CI 64–73); *P* < 0.01]. Intra-aortic balloon pump was present in 4 % (*n* = 6) of days with bleeding compared with 1 % (*n* = 12) of days without bleeding. Type of ECMO was also associated with bleeding (Table 2).

**Table 2:** Comparison of biological and transfusion characteristics of days on ECMO with and without bleeding events

Variable^a^	ECMO days without bleeding (*n* = 1144)	ECMO days with bleeding (*n* = 203)	*P*
Lowest Hb (g/dL)^b^	93 (90–95)	87 (84–89)	<0.01
Lowest platelet count (×10^9^ cells/L)^b^	125 (115–134)	131 (122–141)	0.15
Highest INR^b^	1.6 (1.5–2.0)	1.9 (1.7–2.0)	0.14
Lowest Fg (g/L)^b^	4.0 (3.8–4.3)	3.9 (3.7–4.2)	0.59
Highest aPTT (s)^b^	71 (67–76)	90 (81–98)	<0.01
Lowest arterial pH^b^	7.35 (7.33–7.36)	7.30 (7.28–7.32)	<0.01
Lowest temperature (°C)^b^	36.1 (35.9–36.2)	36.1 (35.9–36.2)	0.85
Highest urea (mmol/L)^b^	11.1 (10.4–11.7)	11.4 (10.6–12.2)	0.40
Lowest corrected calcium (mmol/L)^b^	1.09 (1.07–1.10)	1.02 (1.01–1.05)	<0.01
Highest free haemoglobin^b^	0.07 (0.06–0.08)	0.07 (0.05–0.10)	0.65
RRT^b^	461 (40 %)	76 (37 %)	0.43
Bilirubin^b^	22.6 (19.6–26.0)	22.1 (20.0–24.4)	0.67
*Anticoagulation*			
Heparin infusion^b^	816 (71 %)	106 (52 %)	<0.01
Hours on heparin per day^b^	22 (19–24)	18 (15–20)	0.04
Highest aPTT if receiving heparin (s)^b^	69 (64–73)	86 (76–95)	<0.01
Daily dose heparin if receiving heparin (unit)^b^	19,256 (17,292–21,219)	21,400 (19,187–23,614)	0.06
Centre Alfred Hospital	1085 (95 %)	181 (89 %)	0.10
Max membrane gradient (mmHg)	21 (19–23)	20 (17–22)	0.55
VA ECMO	737 (64 %)	164 (81 %)	<0.01
Type of access if VA ECMO, peripheral	697 (94 %)	146 (89 %)	0.04

*Hb* haemoglobin, *INR* international normalised ratio, *Fg* fibrinogen, *aPTT* activated partial thromboplastin time, *RRT* renal replacement therapy, *IABP* intra-aortic balloon pump, *VA* ECMO veno-arterial ECMO

^a^Mean (95 % CI) adjusted for repeated measures presented for continuous variables and number (%) for categorical variables. *P* values are adjusted for repeated measures

^b^Values are for day prior to bleeding event when considering ECMO days with bleeding

Four patients had an ischaemic stroke (2.7 %), limb ischaemia occurred in 11 patients (7.4 %), and membrane was changed for 16 circuits. There was no difference in thrombotic events between bleeding and non-bleeding patients. None of the patients who received tranexamic acid or activated factor VII were diagnosed with thrombotic complications.

### Factors associated with bleeding

After adjusting for repeated measures in the same patient, factors that were significantly associated with increased risk of bleeding were: higher aPTT on the day prior, with a significant association for the highest quartile compared to the lowest quartile, higher APACHE III score and post-surgical ECMO. Variables associated with lower risk of bleeding were anticoagulation on the day prior to the event (Table 3). ECMO type was not associated with the risk of bleeding in the adjusted model. When considering only the 75 non-post-surgical VA ECMO episodes, higher aPTT on the day prior remained associated with bleeding [adjusted OR 3.24 (95 % CI 1.11–9.44); *P* = 0.03] (Table 4). In this subgroup analysis, the daily lowest temperature was also associated with bleeding occurrence.

**Table 3:** Factors independently associated with bleeding in multivariable analysis

Variable	Adjusted odds ratio	95 % confidence interval	*P*
Previous-day aPTT^a^			
≥46 and ≤55 s	1.35	0.73–2.49	0.33
≥56 and ≤69 s	1.45	0.75–2.82	0.26
≥70 s	3.00	1.64–5.47	<0.01
Previous-day anticoagulation	0.40	0.24–0.66	<0.01
APACHE III score	1.01	1.01–1.02	0.01
Post-surgical ECMO	3.04	1.62–5.69	<0.01

Analysis includes 1125 days with complete data

*aPTT* activated partial thromboplastin time, *APACHE III* score Acute Physiology and Chronic Health Evaluation III score

^a^aPTT < 46 s is the reference

**Table 4:** Factors independently associated with bleeding in non-post-surgical VA ECMO (*N* = 75) (adjusted for ECMO indication and severity)

Variables	Adjusted odds ratio	95 % confidence interval	*P*
*Previous-day aPTT* ^a^			
≥46 and ≤55 s	2.03	0.68–6.10	0.30
≥56 and ≤69 s	1.32	0.41–4.27	0.47
≥70 s	3.24	1.11–9.44	0.03
Previous-day anticoagulation	0.21	0.06–0.52	<0.01
Daily lowest corporeal temperature	1.79	1.20–2.66	<0.01
APACHE III score	1.03	1.01–1.04	<0.01

*aPTT* activated partial thromboplastin time, *APACHE III* score Acute Physiology and Chronic Health Evaluation III score

^a^aPTT < 46 s is the reference

### Independent predictors for survival

Bleeding was associated with worse survival [HR 3.04 (95 % CI 1.58–5.89); *P* < 0.01] on univariate analysis. When adjusting for other variables predictive of survival, bleeding was associated with worse survival [HR 2.17 (95 % CI 1.07–4.41); *P* = 0.03] as given in Table 5. The other variables associated with worse survival were SOFA score at ECMO initiation [HR 1.10 (95 % CI 1.01–1.20); *P* = 0.03] and indication for ECMO (Table 5). When considering only patient undergoing non-post-surgical VA ECMO, bleeding was also associated with worse survival [HR 3.05 (95 % CI 1.11–8.38); *P* = 0.03] (Table 6).

**Table 5:** Factors independently associated with reduced survival

Variables	Adjusted hazard ratio	95 % confidence interval	*P*
Bleeding on one or more days	2.17	1.07–4.41	0.03
Hospital	2.14	0.92–4.94	0.08
APACHE III score	0.99	0.98–1.01	0.39
SOFA score prior to ECMO	1.10	1.01–1.20	0.03
*Indication for ECMO (lung transplant reference)*			
AMI	15.40	1.89–125	0.01
Acute cardiomyopathy	2.05	0.18–23.18	0.56
Chronic cardiomyopathy	1.38	0.08–25.06	0.83
Heart transplant	7.26	0.96–55.23	0.06
Cardiac surgery	9.07	1.06–77.28	0.04
Pneumonia	3.68	0.45–30.12	0.22
Other	12.11	1.73–84.41	0.01

*aPTT* activated partial thromboplastin time, *APACHE III* score Acute Physiology and Chronic Health Evaluation III score, *SOFA* Sequential Organ Failure Assessment, *AMI* acute myocardial infarction

## Discussion

Bleeding complications, as defined by ELSO, occurred in more than half of critically ill patients undergoing ECMO and were strongly and independently associated with hospital mortality. The most frequent bleeding sources identified in our study included ECMO cannula, post-cardiothoracic surgery site and ENT. We found that illness severity and higher aPTT on the day prior to bleeding onset were independently associated with bleeding, while anticoagulant administration on the day prior was associated with a lower risk of bleeding. Our results emphasise the importance of further studies on coagulation abnormalities in this population.

**Table 6:** Factors independently associated with reduced survival in the sub group of patients undergoing non-post-surgical VA ECMO (*N* = 75)

Variables	Adjusted hazard ratio	95 % confidence interval	*P*
Bleeding on one or more days	3.05	1.11–8.38	0.03
Hospital	1.41	0.47–3.62	0.47
SOFA score prior to ECMO	1.11	0.98–1.26	0.11
*ECMO indication (AMI reference)*			
Acute cardiomyopathy	0.16	0.03–0.77	0.02
Chronic cardiomyopathy	0.34	0.07–1.71	0.19
Heart transplant	0.86	0.16–4.69	0.86
Other	0.51	0.19–1.42	0.20

*aPTT* activated partial thromboplastin time, *SOFA* Sequential Organ Failure Assessment, *AMI* acute myocardial infarction

### Comparison with available literature

We found that 60 % of ECMO courses were complicated by bleeding, which is higher than previously reported bleeding rates. This difference may be due to differences in bleeding definitions [3, 11]. Bleeding events in ECMO patients, when reported in the literature, are either not clearly defined or reported on the basis of organ site involved, death due to bleeding, requirement for surgical intervention or using only RBC transfusion as a surrogate of bleeding [2, 11, 13]. Extracorporeal Life Support Organization (ELSO) guidelines recently provided definition criteria for bleeding complications [15]. Although this definition has not commonly been used in studies related to bleeding in ECMO, it may allow and facilitate comparison between findings of ours and future studies.

We found that post-surgical bleeding and ECMO cannula were the most common sources of bleeding in patients undergoing ECMO, as previously reported [1, 11, 16]. However, ENT has not commonly been reported as a source of bleeding in this patient population. Severity of ENT bleeding may be difficult to estimate, and its haemodynamic consequences may be less acute than other bleeding sites. Nonetheless, ENT bleeding is important to diagnose because it may require specific investigation for primary haemostatic disorders, such as platelet function abnormalities and von Willebrand disease, and specific treatment including local haemostatic packing [17]. In our study, ICH complicated ECMO in 2.2 % of cases. This figure is comparable to those reported by Paden et al. but lower than those found in other cohorts [5, 11]. ICH may also be underestimated in our study, as CT brain scans and post-mortem examination were not routinely performed.

Risk factors for bleeding previously studied include parameters available at ECMO initiation (i.e. demographics, ECMO characteristics, patient characteristics at ECMO initiation). There are few reports evaluating bleeding risk factors measured during ECMO treatment. A few decades ago, Kasirajan et al. reported an association between ICH and platelet count [14]. In our study, we did not find an independent association between thrombocytopenia and haemorrhage, possibly because the median platelet count remained relatively high and possibly because of the low rate of the ICH. The same authors reported an association between ICH and heparin therapy. However, information about heparin regimen and coagulation results were not provided [14]. In our study, the fact that a lower proportion of patients with bleeding had received heparin on the previous day compared to those without bleeding may be explained if the onset of bleeding was prior to when the patients met the ELSO bleeding definition, resulting in clinicians ceasing heparin infusion. It could also be explained by physicians selecting patients at lower risk of bleeding for therapeutic anticoagulation, and that patients who remain on heparin have proven their tolerance to anticoagulation without bleeding. However, we also found that the intensity of anticoagulation (as measured by aPTT) was associated with increased bleeding risk (with increasing OR with increasing aPTT categories), suggesting that high aPTT in anticoagulated patients are deleterious. This supports the need for further research on anticoagulation and risk of bleeding in this population. In a recent prospective observational study using thromboelastography (TEG) to evaluate anticoagulation profile during 32 VV ECMO episodes, heparin therapy and aPTT were associated with a reaction time of >90 min (“flat-line” profile); these results also emphasise the need for optimisation of anticoagulation management in this population [18]. Both ECMO and cardiac surgery lead to decrease in fibrinogen level [7, 19]. Association between low fibrinogen level and bleeding events has been recently reported in paediatrics ECMO and in surgical setting [20, 21]. The key role of coagulation abnormalities has been suggested indirectly in other studies, in which a benefit of carefully monitoring and treating coagulopathy in ECMO patients was found [19, 22].

Patients who undergo VA ECMO have different comorbidities, underlying diseases and anticoagulant requirement compared to patients who undergo VV ECMO. While VA ECMO was associated with more bleeding days in the univariate analysis, this association did not remain in the adjusted analysis; however, we may have been underpowered to detect a difference if one did exist. Likewise, there were some differences in bleeding risk according to diagnosis; however, this did not remain associated in the adjusted analysis. Finally, with a high incidence of post-surgical VA ECMO (32 %), we may have missed an association between type of ECMO and bleeding risk despite the absence of colinearity between these post-surgical ECMO and ECMO type.

### Implications of study findings and future research

This risk of bleeding should be taken into consideration in clinical management, including invasive procedures in this population. Although this is a retrospective study with the limitations secondary to this design, our findings on the association between coagulation abnormalities and bleeding in adults on ECMO highlight the importance of closely monitoring coagulation and bleeding in this population. Having local haemostatic guidelines to manage bleeding and non-bleeding patients undergoing ECMO should be a priority for ECMO centres.

Prospective studies evaluating optimal haemostatic treatment, including blood components, intensity of anticoagulation, use of tranexamic acid, concentrate of coagulation factors and antithrombin III with their effects on bleeding and thromboembolic complications are warranted. Beside coagulation monitoring by thromboelastography might also be beneficial in this high-risk population [18, 23].

It would also be meaningful to perform a larger prospective study considering separately fatal bleeding as an outcome.

### Strengths and limitations

Our work addresses an important issue in the management of patients on ECMO, as bleeding complications remain common and associated with poor prognosis. It studies clinical, biological and therapeutic parameters that may be risk factors for bleeding complications. We used a clear definition of bleeding events, thereby ensuring accuracy of our outcome measure and allowing comparisons with other studies. Inclusion of patients from two ECMO centres provides external validity to our results. Time-dependent survival analysis enables identification of parameters not only at ECMO initiation but also during the ECMO course.

Our study does, however, suffer several limitations. Due to the retrospective study design, we could only explore associations between risk factors and bleeding outcomes and cannot make any conclusions regarding causation. We also cannot exclude the possibility of confounding by unmeasured factors, for example cannulation method. The retrospective design may also lead to misclassification of bleeding events. However, the definition of bleeding that we used relies on transfusion volumes, bleeding at specific sites and/or requirement for intervention, all of which could be objectively measured from electronic laboratory data and medical records. As it was not possible to identify the time at which bleeding started, we analysed association between variables collected on the preceding day. Furthermore, we could not perform relevant statistical analysis considering subgroups of either patients undergoing VV ECMO only or post-surgical ECMO because of their small sample size. Finally, thrombosis complications are likely to be underreported because of the retrospective design.

## Conclusions

In conclusion, our study shows that bleeding events as defined by the ELSO remain a common and serious complication in patients undergoing ECMO, with 60 % of ECMO courses complicated by at least one bleeding complication. Bleeding while on ECMO is independently associated with hospital mortality. Among identified risk factors for bleeding event, besides patient severity, coagulation anomalies are the only identified which may be targeted with interventions. These findings highlight the importance of further research to determine the safest haemostatic management in this population.
